# Association Study on *IL4*, *IL13* and *IL4RA* Polymorphisms in Mite-Sensitized Persistent Allergic Rhinitis in a Chinese Population

**DOI:** 10.1371/journal.pone.0027363

**Published:** 2011-11-07

**Authors:** Mei-Ping Lu, Ruo-Xi Chen, Mei-Lin Wang, Xin-Jie Zhu, Lu-Ping Zhu, Min Yin, Zheng-Dong Zhang, Lei Cheng

**Affiliations:** 1 Department of Otorhinolaryngology, The First Affiliated Hospital, Nanjing Medical University, Nanjing, China; 2 Department of Molecular and Genetic Toxicology, School of Public Health, Nanjing Medical University, Nanjing, China; 3 International Centre for Allergy Research, Nanjing Medical University, Nanjing, China; University of Medicine and Dentistry of New Jersey, United States of America

## Abstract

**Background:**

The *IL4*, *IL13*, and *IL4* receptor α chain (*IL4RA*) genes are candidate genes for atopic diseases. We hypothesized that the polymorphisms in these genes are associated with persistent allergic rhinitis (PER).

**Objective:**

To investigate the association of the potential functional polymorphisms in *IL4*, *IL13*, and *IL4RA* with PER induced by house dust mites in a Chinese population.

**Methods:**

Using the TaqMan method, we genotyped six single nucleotide polymorphisms (SNPs) including C-590T in *IL4*, C-1055T and Arg130Gln in *IL13*, and Ile50Val, Ser478Pro and Gln551Arg in *IL4RA*, in a case-control study of 265 patients with PER and 275 healthy controls.

**Results:**

We found that the CT/CC genotypes in *IL4* C-590T were associated with a significantly decreased risk of mite-sensitized PER [adjusted odds ratio (OR)  = 0.64, 95% confidence interval (CI) 0.45–0.92], compared to the TT genotype. Furthermore, PER patients with CT/CC genotypes had significantly lower serum levels of total IgE than those with TT genotype (*P* = 0.001). However, there was no significant association of the *IL13* and *IL4RA* polymorphisms with mite-sensitized PER (*P*>0.05).

**Conclusions:**

Our results suggest that the C-590T polymorphism in *IL4* may contribute to the susceptibility to mite-sensitized PER in a Chinese population.

## Introduction

Allergic rhinitis (AR) is a major public health problem, with a prevalence between 9–24% among the general population [1]. AR is characterized by high serum levels of IgE, overproduction of T helper type 2 (Th2) cytokines, and selective eosinophil accumulation in the nasal mucosa [2]. The development of AR entails a complex interaction between genetic susceptibility and environmental exposure to different factors, of which the most important is the implicated allergen [3], [4]. Although AR has been studied extensively, the mechanism of AR is still not well characterized.

Many candidate genes associated with AR have been identified using position cloning and linkage analysis techniques [5], [6]. Linkage has been reported between serum total IgE levels and chromosome 5q31.1, in which the genes encoding interleukin-4 (IL-4) and IL-13 are involved in the IgE-mediated inflammatory process [7]. IL-4 and IL-13 cytokines are produced by Th2 cells and are capable of inducing isotype class switching of B cells to produce IgE [8]. It has been identified that IL-4 and IL-13 share a common signaling pathway in binding heterodimer of the IL-4 receptor α chain (IL-4Rα) [9]. The IL-4, IL-13, and IL-4Rα interaction pathway have been implicated in the pathogenesis of AR and asthma [10], [11]. In the case of *IL4* gene polymorphism, C-590T (rs2243250), locating in the promoter region, has been shown to be associated with asthma [12], [13]. With respect to the *IL13* gene, two single nucleotide polymorphisms (SNPs) including C-1055T (rs1800925) in the promoter region and Arg130Gln (rs20541) in the exon 4 were reported to be associated with asthma [14], [15], [16]. In addition, three SNPs in the *IL4RA* exons, Ile50Val (rs1805010), Ser478Pro (rs1805015) and Gln551Arg (rs1801275), have been widely investigated for the effects on serum IgE levels and atopy [17].

In the present study, we hypothesized that the potential functional polymorphisms in *IL4*, *IL13*, and *IL4RA* may contribute to the susceptibility to persistent allergic rhinitis (PER). To test this hypothesis, we performed a genotyping analysis for C-590T in *IL4*, C-1055T and Arg130Gln in *IL13*, and Ile50Val, Ser478Pro and Gln551Arg in *IL4RA* in a Chinese population.

## Materials and Methods

### Study subjects

The study included 265 patients with PER and 275 healthy controls. All subjects were recruited in an ongoing study at the First Affiliated Hospital of Nanjing Medical University (Nanjing, China) between May 2008 and January 2011. Our ongoing study was a hospital-based case-control study, which focused mainly on PER patients starting in May 1, 2008. The study aimed to identify genetic markers associated with PER in the Chinese population. The diagnosis of PER was based on the ARIA (2008) guidelines [11]. The selected patients were PER sensitized to house dust mites including *Dermatophagoides pteronyssinus* (*Der p*) and *Dermatophagoides farinae* (*Der f*). Approximately 95% of the eligible patients contacted chose to participate. All controls were recruited from the annual physical exams, who had no clinical features or family history of atopic diseases, and exhibited negative allergen-specific IgE in serum. The control subjects were frequency-matched to the cases by age (±5 years) and sex. The Phadiatop tests were performed in control group. When the serum specific IgE is greater than 0.35 kUA/L, the control subject would be excluded from the study. The response rate of those control subjects we approached for participation in the study was >85%. After interview, 5 mL of peripheral blood sample was collected from each subject. This study was approved by the ethical review board of Nanjing Medical University, and written informed consent was obtained from all participants.

### Allergy testing

The serum total IgE, specific IgE, and eosinophil cationic protein (ECP) levels were measured with an ImmunoCAP100 system (Phadia, Uppsala, Sweden). Total IgE and ECP were determined in all subjects. Specific IgE antibodies to common inhalant allergens including *Der p* (d1), *Der f* (d2), cat epithelium and dander (e1), dog dander (e5), *Blatella germanica* (i6), *Alternaria alternate* (m6), *Ambrosia elatior* (w1), and *Artemisia vulgaris* (w6) were determined in patients with PER.

### Genotyping

Genomic DNA was isolated from leucocytes of venous blood by proteinase K digestion and phenol/chloroform extraction. Genotyping was performed with the TaqMan SNP Genotyping Assay using the 384-well ABI 7900HT Real-Time PCR System (Applied Biosystems, Foster City, CA, USA). For the TaqMan assay, both PCR primers and MGB TaqMan probes are shown in Table 1. Genotype analysis was performed by two persons independently in a blind fashion. About 10% of the samples were randomly selected for repeated genotyping for confirmation, and the results were 100% concordant. The genotyping success rates ranged between 96.0% and 100%.

**Table 1:** Primers and probes for genotypes screening by TaqMan allelic discrimination.

SNPs	NCBIrs No.	Base change	Primers	Probes
*IL4* C-590T	rs2243250	T>C	F: 5′-GGCCTCACCTGATACGACCT-3′	C allele: 5′-FAM-AACATTGTCCCCCAGTG-MGB-3′
			R: 5′-AGAGGCAGAATAACAGGCAGACT-3′	T allele: 5′-HEX-AACATTGTTCCCCAGTGC-MGB-3′
*IL13* C-1055T	rs1800925	C>T	F: 5′-CAACACCCAACAGGCAAATG-3′	T allele: 5′-FAM-AGGAAAATGAGGGAA-MGB-3′
			R: 5′-CTGCAGAATGAGTGCTGTGGA-3′	C allele: 5′-HEX-AGGAAAACGAGGGAA-MGB-3′
*IL13* Arg130Gln	rs20541	G>A	F: 5′-CTGCAAATAATGATGCTTTCGA-3′	G allele: 5′-FAM-GAGGGACGGTTCAACT-MGB-3′
			R: 5′-CCAGTTTGTAAAGGACCTGCTCT-3′	A allele: 5′-HEX-GAGGGACAGTTCAACTG-MGB-3′
*IL4RA* Ile50Val	rs1805010	C>T	F: 5′-CTAACCCAGCCCCTGTGTCT-3′	C allele: 5′-FAM-TCAGGGACACACGTGT-MGB-3′
			R: 5′-GCCCACAGGTCCAGTGTATAGT-3′	T allele: 5′-HEX-TCAGGGATACACGTGT-MGB-3′
*IL4RA* Ser478Pro	rs1805015	T>C	F: 5′-CGCAGGCAACCCTGCTTA-3′	T allele: 5′-FAM-TTCAGCAACTCCCTGAG-MGB-3′
			R: 5′-GCATCTCGGGTTCTACTTCCTC-3′	C allele: 5′-HEX-CAGCAACCCCCTGAG-MGB-3′
*IL4RA* Gln551Arg	rs1801275	A>G	F: 5′-CTCCGCCGAAATGTCCTC-3′	G allele: 5′-FAM-GGCTATCGGGAGTTT -MGB-3′
			R: 5′-GCCTTGTAACCAGCCTCTCC-3′	A allele: 5′-HEX-TGGCTATCAGGAGTTTG-MGB-3′

SNPs, single nucleotide polymorphisms.

### Statistical analysis

Differences in the distributions of demographic characteristics, selected variables, and frequencies of *IL4*, *IL13* and *IL4RA* genotypes between patients with PER and healthy controls were evaluated by using the ANOVA, Student's *t*-test (for continuous variables) or χ^2^-test (for categorical variables). Hardy-Weinberg equilibrium (HWE) was tested using a goodness-of-fit χ^2^-test. The associations between genotypes and PER were estimated by computing odds ratios (ORs) and their 95% confidence intervals (CIs) from unconditional logistic regression analysis with the adjustment for possible confounders. The results of serum total IgE levels were log transformed to normalize the distribution. In the study, Bonferroni correction for multiple testing was applied. The gene-gene interaction was assessed on a multiplicative scale by including the wild-type genotype and heterozygote/homozygote genotypes of each SNP in the unconditional logistic regression model, and then ORs of interaction and their 95% CIs were estimated. The statistical power was calculated by using the PS software (http://biostat.mc.vanderbilt.edu/twiki/bin/view/Main/PowerSampleSize). *P*<0.05 was considered statistically significant, and all statistical tests were two sided. All of the statistical analyses were performed with Statistical Analysis System software 9.1.3 (SAS Institute, Cary, NC, USA).

## Results

### Characteristics of PER patients and controls

As shown in Table 2, the patients with mite-sensitized PER had a mean age of 20.8 years, including 174 males (65.7%) and 91 females (34.3%), and the control subjects had a mean age of 24.3 years and consisted of 177 males (64.4%) and 98 females (35.6%). There were no significant differences in the distribution of age (*P* = 0.136) and sex (*P* = 0.752) between the cases and controls. The serum levels of total IgE (*P*<0.001) and ECP (*P*<0.001) in patients with mite-sensitized PER were significantly higher than those in healthy controls.

**Table 2:** Distribution of selected variables among cases and controls.

Variables	Cases (*n* = 265)		Controls (*n* = 275)		*P*
	N	%	N	%	
Age (years, mean±SD)	20.8±13.1		24.3±14.2		0.136
Sex					
Male	174	65.7	177	64.4	0.752
Female	91	34.3	98	35.6	
Serum total IgE^a^ (log kU/L, mean±SD)	2.61±0.50		1.35±0.51		<0.001
Allergen-specific IgE^a^ (log kUA/L, mean±SD)					
*Dermatophagoides ptoronyssinus*	1.26±0.79				
*Dermatophagoides farinae*	1.25±0.81				
Eosinophil cationic protein (µg/L, mean±SD)	24.93±32.51		7.23±6.07		<0.001

a Serum levels of total IgE (kU/L) and specific IgE (kUA/L) were log transformed to normalize the distribution.

### Information for the *IL4*, *IL13*, and *IL4RA* polymorphisms

The primary information and allele frequencies observed are summarized in Table 3. All genotyped distributions of control subjects were consistent with those expected from the Hardy-Weinberg equilibrium (*P*>0.05). In addition, the minor allele frequency (MAF) of all the six SNPs was consistent with that reported in the HapMap database.

**Table 3:** Primary information of genotyped SNPs in the *IL4*, *IL13*, and *IL4RA* genes.

SNPs	NCBI rs No.	Location	Base change	MAF			*P* for HWE^b^	Genotyped (%)
				HapMap^a^	Case	Control		
*IL4* C-590T	rs2243250	promoter	T>C	0.222	0.172	0.235	0.166	100%
*IL13* C-1055T	rs1800925	promoter	C>T	0.205	0.162	0.148	0.629	99.4%
*IL13* Arg130Gln	rs20541	exon 4	G>A	0.311	0.333	0.291	0.356	99.4%
*IL4RA* Ile50Val	rs1805010	exon 5	C>T	0.367	0.483	0.473	0.991	99.4%
*IL4RA* Ser478Pro	rs1805015	exon 12	T>C	0.100	0.072	0.071	0.720	99.6%
*IL4RA* Gln551Arg	rs1801275	exon 12	A>G	0.167	0.151	0.158	0.310	98.9%

a MAF from the HapMap databases (http://www.hapmap.org).

b HWE *P* value in the control group.

SNPs, single nucleotide polymorphisms.

### Association between the *IL4*, *IL13*, and *IL4RA* polymorphisms and mite-sensitized PER

As shown in Table 4, only the *IL4* C-590T T allele was associated with a significantly decreased risk of mite-sensitized PER (OR = 0.66, 95% CI 0.48–0.90, *P* = 0.008), even after Bonferroni correction (*P* = 0.048). Specifically, compared with the wild-type genotype TT, the heterozygous CT (OR = 0.67, 95% CI 0.47–0.97), but not the homozygous CC (OR = 0.39, 95% CI 0.13–1.14), was associated with a significantly decreased risk of mite-sensitized PER. Because the variant CC genotype was rare in the study population, we combined the CC genotype with the CT genotype (i.e., CT/CC), assuming a dominant genetic model. We found that a significant decreased risk of mite-sensitized PER was associated with the combined CT/CC genotypes, compared to the TT genotype (OR = 0.64, 95% CI 0.45–0.92). However, no significant association with mite-sensitized PER was identified for the other SNPs examined in this study. In addition, the *IL4* C-590T genotypes did not show a significant multiplicative interaction effect with *IL13* and *IL4RA* genotypes (Table 5).

**Table 4:** Genotype and allele frequencies of the *IL4*, *IL13*, and *IL4RA* polymorphisms among cases and controls.

Genotype	Cases		Controls		Crude OR(95% CI)	Adjusted OR(95% CI) a
	N	%	N	%		
*IL4* C-590T	n = 265		n = 275			
TT	179	67.5	157	57.1	1.00	1.00
CT	81	30.6	107	38.9	0.66 (0.46–0.95)	0.67 (0.47–0.97)
CC	5	1.9	11	4.0	0.40 (0.14–1.17)	0.39 (0.13–1.14)
CT/CC	86	32.5	118	42.9	0.64 (0.45–0.91)	0.64 (0.45–0.92)
C allele^b^					0.65 (0.48–0.90)	0.66 (0.48–0.90)
*IL13* C-1055T	n = 264		n = 273			
CC	188	69.3	197	72.2	1.00	1.00
CT	75	28.4	71	26.0	1.14 (0.78–1.67)	1.13 (0.77–1.66)
TT	6	2.3	5	1.8	1.30 (0.39–4.32)	1.25 (0.37–4.19)
CT/TT	81	30.7	76	27.8	1.15 (0.79–1.66)	1.14 (0.78–1.65)
T allele^b^					1.14 (0.81–1.59)	1.13 (0.80–1.58)
*IL13* Arg130Gln	n = 264		n = 273			
GG	114	43.2	134	49.1	1.00	1.00
AG	124	47.0	119	43.6	1.23 (0.87–1.76)	1.23 (0.86–1.75)
AA	26	9.8	20	7.3	1.54 (0.82–2.90)	1.52 (0.80–2.88)
AG/AA	150	56.8	139	50.9	1.27 (0.90–1.78)	1.26 (0.90–1.78)
A allele^b^					1.23 (0.94–1.61)	1.23 (0.94–1.61)
*IL4RA* Ile50Val	n = 264		n = 273			
CC	65	24.6	76	27.9	1.00	1.00
CT	143	54.2	136	49.8	0.97 (0.60–1.59)	1.23 (0.82–1.84)
TT	56	21.2	61	22.3	1.09 (0.67–1.77)	1.07 (0.66–1.57)
CT/TT	199	75.4	197	72.1	1.18 (0.80–1.74)	1.17 (0.80–1.73)
T allele^b^					1.05 (0.82–1.33)	1.04 (0.81–1.33)
*IL4RA* Ser478Pro	n = 265		n = 273			
TT	229	86.4	235	86.1	1.00	1.00
CT	34	12.8	37	13.5	0.95 (0.58–1.57)	0.96 (0.58–1.59)
CC	2	0.8	1	0.4	2.07 (0.19–23.0)	1.99 (0.18–22.1)
CT/CC	36	13.6	38	13.9	0.97 (0.60–1.59)	0.98 (0.60–1.60)
C allele^b^					1.00 (0.63–1.59)	1.01 (0.64–1.61)
*IL4RA* Gln551Arg	n = 261		n = 273			
AA	188	72.0	196	71.8	1.00	1.00
AG	67	25.7	68	24.9	1.02 (0.69–1.50)	1.03 (0.70–1.53)
GG	6	2.3	9	3.3	0.69 (0.24–1.97)	0.69 (0.24–1.98)
AG/GG	73	28.0	77	28.2	0.99 (0.68–1.44)	1.00 (0.69–1.46)
G allele^b^					0.96 (0.69–1.33)	0.97 (0.70–1.34)

a Adjusted for age and sex in logistic regression model.

b Additive model.

**Table 5:** Interaction effect between the *IL4*, *IL13* and *IL4RA* genotypes among cases and controls.

	Interaction with *IL4* C-590T	
SNPs a	OR (95% CI) b	*P* (multiplicative)
*IL13* C-1055T	1.07 (0.50–2.31)	0.865
*IL13* Arg130Gln	0.79 (0.39–1.61)	0.523
*IL4RA* Ile50Val	0.49 (0.22–1.09)	0.081
*IL4RA* Ser478Pro	0.65 (0.23–1.78)	0.398
*IL4RA* Gln551Arg	1.30 (0.59–2.83)	0.514

a SNPs were classified as wild-type genotype and heterozygote/homozygote genotypes. The wild-type genotypes of *IL4* C-590T, *IL13* C-1055T, *IL13* Arg130Gln, *IL4RA* Ile50Val, *IL4RA* Ser478Pro, and *IL4RA* Gln551Arg were TT, CC, GG, CC, TT, and AA, respectively, and the heterozygote/homozygote genotypes of these six SNPs were CT/CC, CT/TT, AG/AA, CT/TT, CT/CC, and AG/GG, respectively.

b Adjusted for age and sex in logistic regression model.

### Association between the *IL4*, *IL13*, and *IL4RA* polymorphisms and serum levels of total IgE, allergen-specific IgE, and ECP

As shown in Table 6, there was a significant association of serum total IgE levels with mite-sensitized PER patients with different genotypes in *IL4* C-590T (*P* = 0.003). Specifically, serum total IgE levels in PER patients with the TT genotype were significantly higher than those with the CT as well as the CT/CC genotypes (*P* = 0.001). No significant differences were found in serum total IgE levels between PER patients with the CC genotype and those with the CT genotype (*P* = 0.658). In addition, no significant differences were found in serum total IgE levels among PER patients with different genotypes in *IL13* and *IL4RA* (*P*>0.05, data not shown). Furthermore, there was no significant association of serum specific IgE or ECP levels with mite-sensitized PER patients with different genotypes in *IL4*, *IL13*, and *IL4RA* (*P*>0.05, data not shown). Similarly, no significant differences were found in serum total IgE and ECP levels among control subjects with different genotypes in *IL4*, *IL13*, and *IL4RA* (*P*>0.05, data not shown).

**Table 6:** Association between the *IL4* C-590T genotypes and serum total IgE levels in patients with mite-sensitized persistent allergic rhinitis.

Genotypes	N	%	Total IgE a (log kU/L, mean ± SD)	*P* b *P* c
TT	179	67.5	2.68±0.49	0.003
CT	81	30.6	2.47±0.51	0.001
CC	5	1.9	2.37±0.17	0.161
CT/CC	86	32.5	2.46±0.50	0.001

a Serum levels of total IgE (kU/L) were log transformed to normalize the distribution.

b Analysis by ANOVA among TT, CT and CC genotypes.

c Compared with the TT genotype by Student's *t*-test.

## Discussion

In the present case-control study, we explored the association between the *IL4*, *IL13*, and *IL4RA* polymorphisms and mite-sensitized PER in a Chinese population. We found, for the first time, that the C-590T polymorphism in the *IL4* promoter region was significantly associated with PER induced by house dust mites.

Until now, it is clear that no single genetic risk factor is responsible for the development of AR. However, cumulative evidence has suggested that the development of AR in an individual will depend on the interaction of a number of genes and various environmental factors [18]. The review by Davila et al. suggested that AR patients have involved genes encoding for cytokines and their receptors, due to the implication of many cytokines in the pathogenesis of AR [3]. Cytokines play a crucial role in the widely used immunological model that explain the increasing prevalence of atopic diseases by an altered balance between Th1 and Th2 immune response triggered by lifestyle-determined changes in environment [19]. IL-4 is a typical Th2 cytokine of decisive significance in regulating Th1/Th2 balance [20]. For IL-4, the encoded protein is a cytokine produced by activated T-cells and a ligand for the IL-4RA. IL-4R binds not only IL-4, but also IL-13. IL-4 induces immature effector cells to assume a Th2 phenotype and also repress Th1-inducing signals [21]. Rosenwasser et al. [22] reported a functional polymorphism C-590T in the promoter region of *IL4*, and the T allele was associated with an increased *IL4* gene expression *in vitro*. Such a functional polymorphism in the *IL4* gene may elevate IL-4 levels and thereby influences the IL-4 dependant events which determine disease progression [23]. Several recent studies have demonstrated an association of the T allele with asthma and atopy in different populations and ethnic groups [13], [24]. In the present study, we found that mite-sensitized PER patients with the CT/CC genotypes in *IL4* C-590T had significantly lower serum total IgE levels than those with the TT genotype. Furthermore, the CT/CC genotypes were associated with a significantly decreased risk of mite-sensitized PER. Our results suggested that individuals with the CT/CC genotypes had a protective role for mite-sensitized PER, which was in accordance with the previous findings in asthma [12], [13]. Failure to adjust for multiple testing appropriately may produce excessive false positives, or overlook true positive signal [25]. In our study, the association after Bonferroni correction for multiple testing still remained statistical significance, suggesting that our findings were quite unlikely due to statistical chance.

In previous studies, the C-1055T and Arg130Gln polymorphisms in the *IL13* gene have been associated with total serum IgE levels, atopy, asthma and AR [8], [18], [26]. Functional studies support a regulatory role for the C-1055T variant allele and suggest that the 130Gln substitution results in signal transducer and activator of transcription 6 (STAT6) phosphorylation in monocytes, decreased affinity of IL-13 for IL-13 receptor α-2, and increased expression of IL-13 [4], [27]. Specially, a number of studies were conducted to investigate the association between *IL13* Arg130Gln polymorphism and atopic diseases. But these studies reported conflicting results. Many studies have found that there was a significant association with increased serum total IgE levels in asthma patients [28], [29], [30], whereas others failed to confirm such findings [8], [31]. Wang et al. [32] reported that *IL13* Arg130Gln polymorphism was not associated with mite-sensitized PER and *Artemisia* pollinosis in a Chinese population from Northern China. In a previous study, we also showed that there was no relationship with *IL13* Arg130Gln polymorphism involved in the development of cedar pollinosis in a Japanese population [33]. In the current study, we failed to detect significant association between *IL13* polymorphisms and susceptibility to mite-sensitized PER in a Chinese population from Eastern China. Although it is also biologically plausible that other functional polymorphisms of *IL13* are involved in the development of AR, susceptibility may be different because of diverse ethnic differences in environment exposure and sequence variants. However, this finding needs to be validated by other ethnic populations.

The *IL4RA* gene is a candidate gene for involvement in atopy, asthma and AR [14], [34]. Functional studies have shown that Ile50Val, Ser478Pro, and Gln551Arg polymorphisms in the exons of *IL4RA* have functional consequences such as changes in transcription rates, enhanced activity or signaling through the protein, or changes in serum protein levels in diverse cell types [10], [35], [36], [37]. The variants in *IL4RA* have been reported to be involved in the risk for hyper-IgE syndrome, atopic dermatitis, and the asthma phenotype [38]. Nakamura et al. [39] found significant differences in the frequencies of Ile50Val and Glu375Ala polymorphisms in *IL4RA* between patients with cedar pollionsis and healthy subjects in a Japanese population. Nevertheless, we did not observe significant association of Ile50Val, Ser478Pro, and Gln551Arg polymorphisms with mite-sensitized PER in our study. The reason for these different findings remains unclear, which might due to ethnically diverse and/or miscellaneous environmental factors.

Several limitations of the present study should be addressed. Because our study was a hospital-based study design, the study subjects may not be representative of the general population. However, the agreement with Hardy-Weinberg equilibrium suggested that the selection bias in terms of genotype distribution would not be substantial. In addition, relatively small sample size (265 cases and 275 controls) may influence the statistical power in the study. However, we have 80% power at 0.05 significance level to detect an OR of 1.50 or higher and 0.62 or lower with an exposure frequency of 20% under the current sample size. Therefore, large population-based prospective studies with ethnically diverse populations are warranted to further elucidate the impact of *IL4*, *IL13*, and *IL4RA* polymorphisms on AR susceptibility.

In conclusion, our present study suggests that the C-590 T polymorphism in the *IL4* gene is associated with susceptibility to PER induced by house dust mites, but the functional relationship still needs clarification.
